# Therapeutic adjuncts in the endoscopic management of urethral stricture disease: past, present, and future

**DOI:** 10.3389/fruro.2024.1342941

**Published:** 2024-03-27

**Authors:** Jas Singh

**Affiliations:** Division of Urology, McGill University, Montreal, QC, Canada

**Keywords:** urethral stricture, therapeutic, injection, reconstruction, endoscopy

## Abstract

Urethral stricture disease is a recurrent and debilitating condition affecting many men of all ages. Management may involve endoscopic or surgical treatment. Surgical urethral reconstruction remains the gold standard treatment and is associated with higher success rates in terms of stricture recurrence free-survival. However, urethroplasty is not available to patients with significant medical comorbidities, or those wishing to forego invasive surgery. Endoscopic treatment is aimed at improving lower urinary tract symptoms and relieving obstruction while maximizing time to stricture recurrence with the aid of therapeutic adjuncts. The aim of this review is to discuss the mechanism of action and role of therapeutic adjuncts and highlight some of the lesser-known adjuncts that have been utilized with success in this space.

## Introduction

Urethral stricture disease (USD) is a recurrent and debilitating condition affecting approximately 0.6% of all men with increasing prevalence among men over the age of 65. In addition, the rate of USD in the population has been increasing since the early 2000s (1). In addition to the morbidity of a recurrent pathologic process with repeated instrumentation and treatments, the health care expenditure related to USD treatment has been substantial with estimates of up to 200 million dollars per year in the United States (2). Patients who are not suitable for surgical reconstruction or who wish to pursue less invasive options first, may be offered endoscopic treatment with or without adjunct therapeutic treatment. A recent systematic review and meta-analysis reported that any form of adjunct use was associated with a lower rate of USD recurrence compared with no adjunct use. The reintervention rate was 44.1% to 61.9% in the control group and 7% to 70.6% in the adjunct group (3).

The endoscopic management of USD may include several different techniques for achieving stricture dilation or disruption. These include intermittent urethral dilatation with coaxial urethral dilators and sounds, direct vision internal urethrotomy (DVIU), and pressure balloon dilation. The local delivery of therapeutic adjuncts may be performed using intraurethral instillation, intralesional injection, drug-coated urethral catheters, or drug-coated balloons (4).

In this review, we aim to discuss past, present, and potential future therapeutics adjuncts for endoscopic USD management.

## Past therapeutic adjuncts

### Captopril

Captopril is an angiotensin-converting enzyme inhibitor that has antifibrotic properties. It has been used to inhibit fibroblast proliferation in irradiated lung tissue and in paraquat-induced pulmonary fibrosis (5, 6). Captopril has been evaluated as an antifibrotic adjunct following ureteral injury in a rabbit model.

Pan et al., evaluated the role of captopril following ureteral injury in New Zealand rabbits. First, the rabbits underwent surgical induction of ureteral injury to establish the ureteral injury animal model. Two weeks after injury, the injured ureteral segment was harvested along with a segment of normal contralateral ureter to serve as the control. Using reverse-transcriptase polymerase chain reaction, the expression of epidermal growth factor (EGF), TGF-β, keratosis growth factor (KGF), type I collagen a1, type I collagen a2, type III collagen (COL III) and fibronectin (FN) were evaluated. The authors noted significantly greater expression of EGF, TGF-β, FN, Col Ia1 and Col Ia2 in the injured ureteral segment compared with the normal contralateral ureter. After establishing this baseline, 10 male adult New Zealand rabbits were randomly assigned to the captopril group or the control group after constructing the ureteral injury models. In the treatment group, 10 mg/kg/day of captopril was given orally while placebo was used for the control group. In the captopril group, the elevation of EGF, FN, and Col Ia2 were significantly decreased compared to the control group (p < 0.05). However, TGF-β and Col Ia1 did not show a significant difference. Histologically, there was less fibrotic scarring and less collagen deposition in the smooth muscle layer and ureteral serosa in the captopril group (7).

The only in human study of captopril for recurrence USD was reported by Shirazi et al., in 2007. After conducting a phase I evaluation of tolerability to side effects in 13 rabbits, a phase II study was performed on 56 men. Following DVIU, patients were allocated to one of three groups in which an intraurethral gel was instilled. The groups included a placebo group, a 0.1% captopril gel group and a 0.5% captopril gel group. Most strictures were between 1 and 2 cm (41.1%). At a mean follow-up of 16 months, the maximum urinary flow rate was higher in both captopril treatment groups compared with the placebo group. The stricture recurrence rate was also significantly lower in both treatment groups (p < 0.05). No significant differences in urine flow rate and recurrence were reported between the treatment groups (8).

Summary: Captopril has been used with success in a single human study. However, additional studies are needed to confirm safety and efficacy.

### Intraurethral brachytherapy

In 2001, Sun et al., reported their experience with intraurethral brachytherapy in a small sample of 17 patients with recurrent urethral strictures. All patients underwent internal urethrotomy or transurethral scar resection followed by catheter-based intraurethral brachytherapy with 192-iridium in two separate dosages for a total of 1000 to 1500 cGy. At a mean follow-up of 20 months, 16/17 (94.1%) patients were stricture free. The single case of recurrence occurred at 3 months follow-up. No significant complications were reported. This early case series is the only reported study utilizing this technique for recurrent urethral strictures (9).

Summary: Only a single study exists on a small sample of patients for this technique. Additional studies are warranted.

### Botulinum toxin

Botulinum toxin (BoNT) is a neurotoxin produced by a Gram-positive, rod-shaped, anaerobic bacterium called *Clostridium botulinum*. There are seven immunologically distinct neurotoxin subtypes, but the most clinically utilized and relevant is onabotulinum toxin A (BoNT-A). BoNT-A induces neuromuscular blockade by the inhibition of acetylcholine release from the presynaptic cholinergic neuromuscular junction (10). The end result is the chemoimmobilization of striated or smooth muscle. BoNT has been used extensively in lower urinary tract dysfunction including neurogenic and non-neurogenic detrusor overactivity and external urethral sphincterotomy for patients with detrusor-sphincter dyssynergia (11, 12).

It has been hypothesized that repeated displacement of injured tissue leads to an enhanced inflammatory response during wound healing and acceleration of scar formation. Furthermore, the added tensile force induced by muscle fibers contracting during wound healing can result in significant contraction on immature collagen. As a result, it has been theorized that temporary muscle paralysis may help minimize tension at the level of the healing wound edges. There is evidence within the dermatologic and plastic reconstructive surgical literature that the fibrotic response to tissue injury can be attenuated by the injection of BoNT at the level of the scar to prevent or reduce the tension effects of muscle contraction on wound edges during healing (13, 14). Urethral anatomy consists of urothelium, lamina propria, and smooth muscle fibers. Therefore, paralysis of the smooth muscle fibers using BoNT may help reduce wound contraction during tissue healing following internal urethrotomy or stricture dilation.

In order to evaluate this hypothesis, Sahinkanat and colleagues, compared urethral fibrosis outcomes in prepuberal male Swiss albino rats divided into two groups. Group one consisted of 20 rats which received BoNT-A injection and group two which consisted of 10 rats who received 0.9% normal saline solution injection, following surgically induced urethral injury. Rats in the treatment group received 1.5 U (0.1 mL) BoNT-A at concentration of 15 U/mL in a periurethral location at 4 points. A similar technique of was utilized for the control group. At 21 days, the rats were killed and the urethral location of injection harvested. The urethral sections were examined histopathologically using a single blinded pathologist. The result was categorized as negative (no fibrosis), slightly positive (<25% fibrosis), moderately positive (25%-50% fibrosis), and very positive (>50% fibrosis). In the control group, severe urethral fibrosis was identified in contrast to only mild fibrotic changes in the BoNT-A group (p < 0.01). In addition, the urethral specimens from the control group exhibited increased fibroblast and collagenase areas compared with the BoNT-A group (15).

In 2004, Khera et al., reported on a small patient series using BoNT-A for recurrent urethral strictures. Three patients with a history of posterior urethral stricture disease following failed DVIU, underwent repeat DVIU with BoNT-A injection. Two of the patients had strictures following radical retropubic prostatectomy for prostate cancer and the third patient had a prior transurethral microwave procedure for BPH. All patients underwent radial incision of the stricture in 4 quadrants followed by injection of 100 U of BoNT-A diluted in 2 mL of preservative-free saline. At 9 months postoperative, two of the three patients had satisfactory urethral patency to allow unobstructed voiding while the third patient was able to maintain patency at 12 months postoperative (16). Given that the clinical effect of BoNT-A reverses within 3 to 6 months, the long-term success of this therapeutic adjunct is undetermined and no further studies exist.

Summary: BoNT-A has demonstrated some success as an adjunct and holds promise given the theorized mechanism of action on the underlying muscle. However, additional data from prospective studies is needed to establish safety, tolerability, and efficacy.

## Present therapeutic adjuncts

### Steroids

Steroids are well known inhibitors of inflammation. The most widely studied steroidal adjuncts for USD are triamcinolone and methylprednisolone. Both intralesional and intraurethral instillation using drug-coated intermittent-self catheterization has been studied. In a systematic review of 8 randomized clinical trials with 203 patients, who were treated with DVIU followed by either local steroid injection or lubricated catheter, steroid injection was reported to be associated with a significantly longer time to stricture recurrence. However, there was no difference between overall rate of recurrence (17). In another systematic review, 7 randomized trials with 365 patients were evaluated in which patients underwent DVIU plus local urethral steroids versus DVIU alone. Local steroid use was associated with a reduced recurrence rate and time-to-recurrence (18). Steroidal adjuncts are readily available and have demonstrated efficacy with minimal side effects and toxicity.

#### Summary

Steroids may be used as a therapeutic adjunct following DVIU, with good evidence.

### Chemotherapeutics

Several chemotherapeutic agents have been utilized for their antifibrotic properties in treating USD. Mitomycin C (MMC) inhibits several cellular processes that contribute to fibrosis including fibroblast proliferation and collagen synthesis (19). In a systematic review and meta-analysis of urethral stricture adjuncts, MMC was found to be associated with the lowest rate of USD recurrence among all adjuncts evaluated (3).

Other agents that have been tested include the taxanes, docetaxel and paclitaxel which have demonstrated antifibrotic properties. These agents function as mitotic inhibitors and stabilizers of cellular microtubules which interfere with normal cellular division and migration. In addition, they inhibit new tissue growth and fibrotic scarring (20).

A recent development utilizing this therapy is the Optilume^®^ drug-coated balloon containing a paclitaxel coating which has demonstrated a strong clinical response in recent trials. At three years of follow up, 77% of patients were free from repeat intervention including a 176% increase in maximum urinary flow and a 65% decrease in International Prostate Symptom Score (20, 21). Properties that have contributed to the success of paclitaxel in the urethral environment include its lipophilic and hydrophobic structure which enables rapid uptake by urothelial cells and limits washout (22).

#### Summary

Mitomycin C is associated with the best results of USD management with respect to stricture-free recurrence compared with other adjuncts. While rare, it is worth noting that severe complications have been reported in the literature including osteitis pubis, urosymphyseal fistula, and rectourethral fistula when used in the treatment of recalcitrant bladder neck stenosis. DVIU and injection at the 6 and 12 o’clock positions should be avoided for these reasons.

### Hyaluronidase

Hyaluronidase is an endoglycosidase, which is an enzyme that is responsible for breaking down the glycosidic bonds within hyaluronic acid. It is also responsible for the breakdown of other mucopolysaccharides in connective tissue (23). Hydase (hyaluronidase injection) was first approved by the United States Food and Drug Administration in 2005 and is approved for use as an adjuvant to increase the absorption and dispersion of other injected drugs, for hypodermoclysis, and as an adjunct in subcutaneous urography for improving resorption of radiopaque agents (24). Aside from its on-label use, hyaluronidase has been used off-label in various urological applications including the reduction of paraphimosis, interstitial cystitis/bladder pain syndrome, and recurrent USD (25–27). In the urethra, hyaluronidase has been utilized for its antifibrotic properties given its ability to suppress inflammatory wound mediators. This includes reducing fibroblast proliferation, collagen deposition, and glycosaminoglycan synthesis (27).

The post DVIU injection of hyaluronic acid (HA) with carboxymethylcellulose (CMC) for recurrent USD has been investigated in two separate studies. The idea for HA/CMC in the urethra comes from the use of this combination of anionic polysaccharides in abdominopelvic surgery (28). It has been reported that human urethral stricture tissue has low levels of HA and high levels of dermatan sulfate, suggesting that intralesional instillation of HA may reduce the possibility of stricture recurrence (29).

In 2010 Kim et al., retrospectively evaluated 17 patients who underwent DVIU followed by Foley catheter placement and peri-catheter injection of a mixed solution of HA/CMC. Patients were evaluated at 3 and 12 months postoperatively for success which was defined as a maximum flow rate of at least 15 ml/s or no visible urethral stricture on retrograde urethrogram. The success rate was 76.5% and 52.9% at 3 and 12 months postoperatively, respectively. The success rate for strictures less than 10 mm was 63.6% and 33.3% for strictures greater than 10 mm in length. The success rate for single strictures was 61.5% and 25% for multiple strictures. The authors concluded that adjunct instillation of HA/CMC following DVIU was no better than DVIU alone in this small sample series (30).

In 2013, Chung et al., reported on a multicenter, single-blinded, randomized controlled trial comparing evaluating HA/CMC injection. A total of 120 patients were evenly randomized following DVIU to either HA/CMC instillation or lubricant instillation with 53 patients and 48 patients completing the treatment in each group, respectively. Patients were evaluated for pain and degree of satisfaction. At a short follow up only 1 month, pain scores were lower in the HA/CMC group and the degree of satisfaction was significantly higher. The urethral stricture recurrence rate was 9.4% in HA/CMC and 22.9% with lubricant instillation alone. From this study, it appears that HA/CMC use following DVIU can help prolong time to stricture recurrence and also assist with post-procedural pain (31).

Hyaluronidase treatment has also been evaluated in combination with other urethral injectable adjuncts. In 2015, Kumar et al., evaluated intralesional injection of a four-drug cocktail consisting of Triamcinolone 40 mg, Mitomycin 2 mg, Hyaluronidase 3000 units, and N-acetyl cysteine 600 mg, diluted in 5-10 mL of saline. A total of 50 patients underwent holmium laser DVIU followed by intralesional injection. Follow up ranged from 6 to 18 months. The overall success rate was 82%. The success rate for strictures <1 cm, 1-3 cm, and >3 cm was 100%, 81.2%, and 66.7%, respectively. Complications were not reported (32).

#### Summary

Hyaluronidase has been used with success, primarily in combination with other adjuncts and may be used for USD treatment. However, additional prospective studies are needed.

## Future therapeutic adjuncts

### Platelet-rich plasma and mesenchymal stem cells

Cellular and non-cellular regenerative therapies have been receiving considerable attention in many different applications due to their tissue regenerative capacity. Platelet-rich plasma (PRP) are concentrated suspensions of platelets that have undergone removal of all other cellular components from whole blood. These suspensions are rich in growth factors and cytokines such as TGF-β, PDGF, and VEGF which are involved in angiogenesis and wound healing (33).

In a randomized controlled trial of 87 male patients with symptomatic bulbar urethral stricture, patients were allocated to DVIU followed by submucosal saline injection or autologous PRP. At 12 months follow-up, stricture rates were 26.82% and 9.09% in the control and PRP groups, respectively. At 2 years follow-up, the recurrence rate in the control and PRP groups was 43.9% and 21.95%, respectively (34).

Adipose derived mesenchymal stem cells (MSC) have been shown to decrease type I and III collagen deposition (35). The anti-fibrotic mechanism of MSC in USD is believed to be secondary to the expression of TNF-α induced exosomal miR-146a expression (36). They are also responsible for decreasing pro-inflammatory cytokines such as TNF-α and IL-1β. Castiglione et al., investigated human adipose tissue-derived stem cells in a rat model and demonstrated a decreased expression of several fibrosis-related genes in the stem cell group compared with rats the controls (37).

Summary: These therapies hold promise in this space, but additional prospective studies are needed.

### Tyrosine kinase and YAP/TAZ pathway inhibitors

Nintedanib is a competitive inhibitor of both nonreceptor tyrosine kinases (nRTKs) and receptor tyrosine kinases (RTKs.). Nintedanib targets VEGF, FGFR, and PDGFR and has been used in the treatment of idiopathic pulmonary fibrosis and non-small cell lung cancer. Recently, Bozkurt et al., reported their experience with Nintedanib in fibrosis after urethral trauma. They allocated 23 adult rats into three groups of sham, urethral injury, and urethral injury with oral Nintedanib for 14 days. After 14 days, the rat urethras were examined histopathologically and immunohistochemically and there was a significant reduction in inflammatory cell infiltration, spongiofibrosis and TGF-β and VEGF levels in the Nintedanib group (38).

Verteporfin is an inhibitor of the YAP/TAZ pathway which is a central signaling pathway in the wound healing process (39). It is currently only approved for use in photodynamic therapy for age-related macular degeneration but has demonstrated activity in fibrosis reduction in several studies including the treatment of Peyronie’s plaques obtained from patients during the time of incision and grafting (40).

Summary: Additional pre-clinical studies are needed with the aim of translation into human studies.

**Table 1** summarizes the aforementioned therapeutic adjuncts.

## Conclusion

The use of therapeutic adjuncts following the minimally invasive treatment of USD has demonstrated significant success beyond endoscopic treatment alone. A myriad of options exists with a multitude of mechanisms of action and treatment delivery. Among the many options, the best evidence supports the use of Mitomycin C as an intralesional injection following DVIU and in some cases steroids. Use should be limited to strictures less than 1.5-2 cm due to declining rates of success for longer strictures. However, the current use of these adjuncts is not specifically endorsed by any international urological society guidelines due to limited long-term follow-up and uncertainty about the optimal drug, dose, volume and delivery technique including off-label use of these agents. Ongoing studies, particularly randomized clinical trials are gravely needed in this space in order to maximize understanding of the factors predicting success, as not all patients with USD will be suitable for surgical reconstruction.

## Author contributions

JS: Conceptualization, Data curation, Writing – original draft, Writing – review & editing.

## Appendix

**Table 1:** Summary of therapeutic adjuncts utilized in endoscopic USD treatment.

AGENT/MODALITY	MECHANISM	SUCCESS RATE	EVIDENCE
**Captopril**	ACE-inhibitor with fibroblast inhibition	Reduced recurrence rate vs placebo	Shirazi et al. (8)
**Intraurethral Brachytherapy**	192-iridium stricture irradiation	94.1% at 20 months	Sun et al. (9)
**Botulinum toxin**	Submucosal muscle paralysis to minimize wound tension	N = 3, 100% at 9 months	Khera et al. (16)
**Steroids**	Inhibition of fibroblasts and collagen deposition	RR = 0.67 (for recurrence) HR = 0.58 (for time-to-recurrence)	Zhang et al. (17) Soliman et al. (18)
**Chemotherapeutics**	Inhibition of fibroblasts and collagen deposition	OR = 0.23	Pang et al. (3)
**Hyaluronidase with** **Carboxymethylcellulose**	Inhibition of fibroblast proliferation, collagen deposition, and GAG synthesis	76.5% at 3 months 52.9% at 12 months < 1cm = 63.6% > 1cm = 33.3%	Kim et al. (30)
**PRP/MSC**	Enhanced wound healing, reduce inflammatory cytokines, reduce collagen deposition	90.9% at 12 months 78% at 2 years	Rezaei et al. (34)
**TK and YAP/TAZ Inhibitors**	Inhibition of VEGF, FGFR, PDGFR, and wound healing process	—	—

ACE, angiotensin-converting enzyme; GAG, glycosaminoglycan; HR, hazard ratio; MMC, mitomycin C; MSC, mesenchymal stem cells; OR, odds ratio; PRP, platelet-rich plasma; RR, risk ratio; TK, tyrosine kinase; USD, urethral stricture disease.
